# Poly[[bis­(μ_4_-acetato-κ^4^*O*:*O*:*O*′:*O*′)tetra­kis­(μ_3_-acetato-κ^3^*O*:*O*:*O*′)bis­(μ_2_-acetato-κ^2^*O*:*O*′)bis­(μ_3_-hydroxido)penta­nickel(II)] 2.60-hydrate]

**DOI:** 10.1107/S2414314625000823

**Published:** 2025-02-07

**Authors:** Maximilian Pfeiffer, Berthold Stöger, Matthias Weil

**Affiliations:** a TU Wien, X-Ray Centre, Getreidemarkt 9/E057, 1060 Vienna, Austria; b TU Wien, Institute for Chemical Technologies and Analytics, Division of Structural Chemistry, Getreidemarkt 9/E164-05-1, 1060 Vienna, Austria; Goethe-Universität Frankfurt, Germany

**Keywords:** crystal structure, nickel, basic acetate, disorder, hydrogen-bonding

## Abstract

The title compound, {[Ni_5_(C_2_H_3_O_2_)_8_(OH)_2_]·2.60H_2_O}_*n*_ or [Ni_5_(OAc)_8_(OH)_2_]·2.60H_2_O (OAc is the acetate anion, C_2_H_3_O_2_), represents a hydrated basic acetate. Its asymmetric unit comprises half of the formula unit, with one of the three unique Ni^II^ cations situated at an inversion centre. The Ni^II^ atoms are in octa­hedral coordination environments by O atoms of the acetato ligands and by the basic OH group. The different kinds of bridging modes (μ_2_, 2×μ_3_, and μ_4_ for the acetato ligands; μ_3_ for the OH group) lead to the formation of a framework structure with hydro­phobic channels extending parallel to the main crystallographic axes. Disordered water mol­ecules are situated in pockets close to the OH groups and are held in place by hydrogen-bonding inter­actions.

## Abstract 2

The basic nickel acetate [Ni_5_(OAc)_8_(OH)_2_]·2.60H_2_O crystallizes in a polymeric framework structure with all three unique nickel cations in octa­hedral coordination by O atoms.

## Graphical abstract

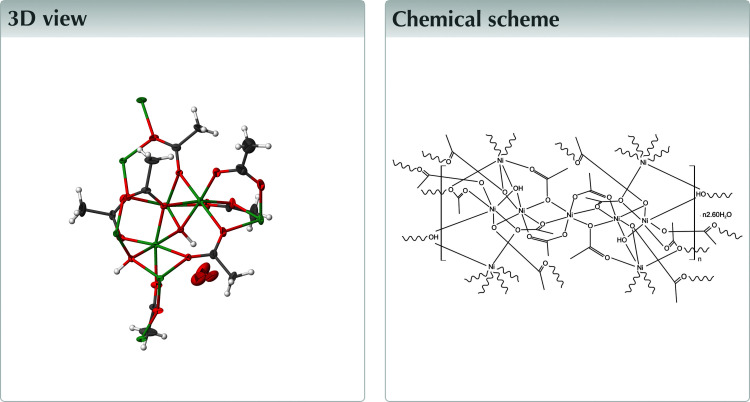

## Structure description

Nickel acetate, Ni(OAc)_2_, is a common precursor for the synthesis of oxygen-containing nickel compounds and is usually employed in form of its tetra­hydrate. As it decomposes easily when the temperature is increased, it is used for typical solid-state reactions. As a result of its good solubility in water, nickel acetate can also be used for syntheses in aqueous media or under hydro­thermal conditions. Precisely for this purpose, Ni(OAc)_2_ was employed as a precursor intended for phase-formation studies of nickel arsenates under hydro­thermal conditions. However, a basic nickel acetate of composition [Ni_5_(OAc)_8_(OH)_2_]·2.60H_2_O had formed serendipitously instead, and its crystal structure is reported here.

In general, basic acetates comprise metal cations bound to a collection of acetate anions and to an O^2–^ ion or an OH^−^ group. The latter bridge several metal atoms (*M*) and thus form oxido-centred coordination polyhedra, usually with {O*M*_3_}-/{(HO)*M*_2_}-trigonal–planar or {O*M*_4_}-/{(HO)*M*_3_}-tetra­hedral shapes. These kinds of structural features are observed, for example, in the acetate compounds Be_4_O(OAc)_6_ (Pauling & Sherman, 1934 ▸), Mg_3_O(OAc)_4_ (Scheurell *et al.*, 2015 ▸), [Cr_8_(OH)_8_(OAc)_16_]·30H_2_O (Eshel & Bino, 2001 ▸), Fe_3_O(OAc)_7_(HOAc) (Abrahams *et al.*, 2024 ▸), Cu_2_(OH)_3_(OAc)·H_2_O (Švarcová *et al.*, 2011 ▸), Pb_3_O_2_(OAc)_2_·0.5H_2_O (Mauck *et al.*, 2010 ▸), Pb_4_O(OAc)_6_ or Pb_2_O(OAc)_2_ (Martínez Casado *et al.*, 2016 ▸). In the title compound, an {(HO)Ni_3_} unit with tetra­hedral shape is present, as discussed in more detail below.

**Figure 1:**
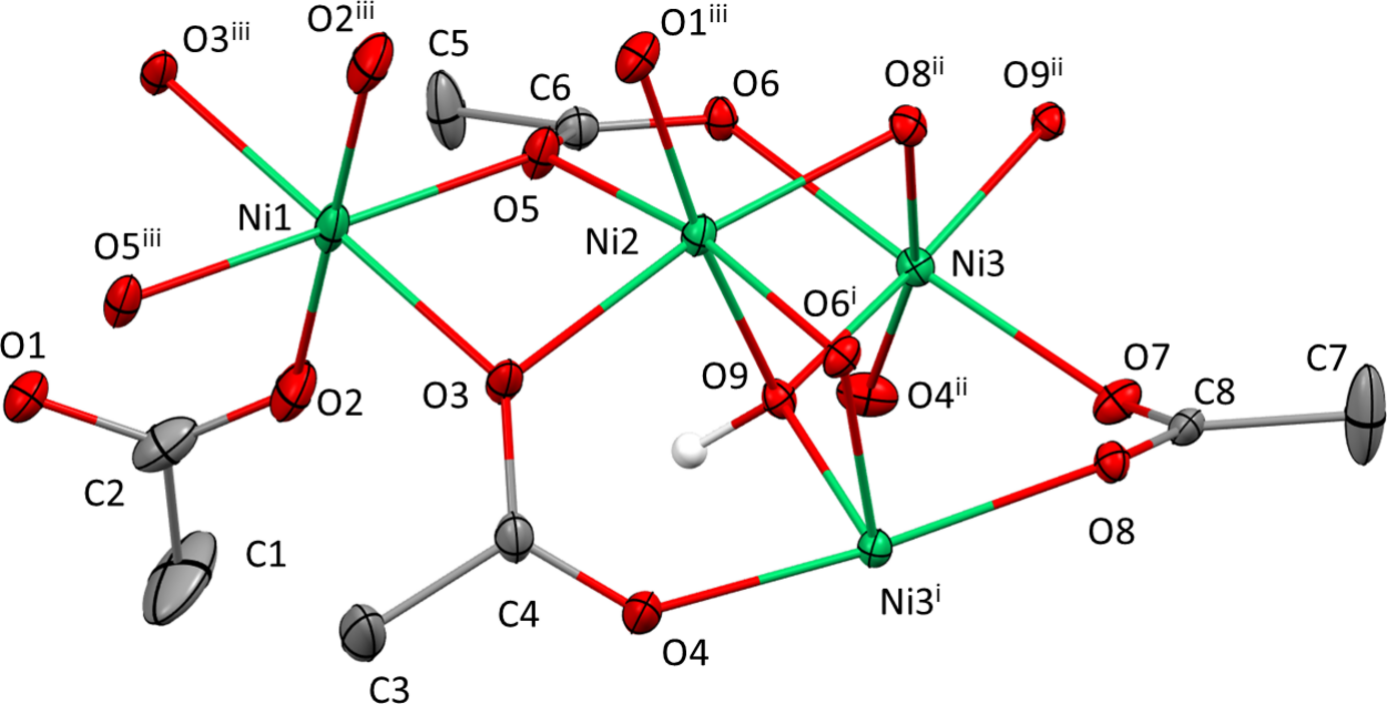
The coordination of the Ni^II^ atoms in the crystal structure of [Ni_5_(OAc)_8_(OH)_2_]·2.6H_2_O. Displacement ellipsoids are drawn at the 40% probability level; for clarity, methyl H atoms and the O atoms of disordered water mol­ecules are not shown. [Symmetry codes: (i) −*y* + 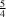, *x* − 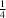, *z * − 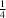; (ii) *y* + 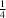, −*x* + 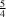, *z* + 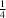; (iii) −*x* + 2, −*y* + 1, −*z* + 1.]

[Ni_5_(OAc)_8_(OH)_2_]·2.60H_2_O is isostructural with the magnesium analogue, [Mg_5_(OAc)_8_(OH)_2_]·1.19H_2_O (Scheurell *et al.*, 2015 ▸). The asymmetric unit of [Ni_5_(OAc)_8_(OH)_2_]·2.60H_2_O comprises of half of the formula unit, with Ni1 situated at a special position (multiplicity 8, Wyckoff letter *d*, site symmetry 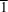) of space group *I*4_1_/*a*. The three Ni^II^ cations are octa­hedrally surrounded by O atoms, with Ni1 only by carboxyl­ate O atoms, Ni2 by five carboxyl­ate O atoms and one O atom (O9) of the OH group, and Ni3 by four carboxyl­ate O atoms and two OH groups (Fig. 1 ▸Table 1 ▸). The Ni—O distances range from 1.993 (2) to 2.1259 (18) Å, with a mean of 2.063 (55) Å, which is close to the literature value of 2.070 (54) calculated for 242 [NiO_6_] polyhedra (Gagné & Hawthorne, 2020 ▸).

**Figure 2:**
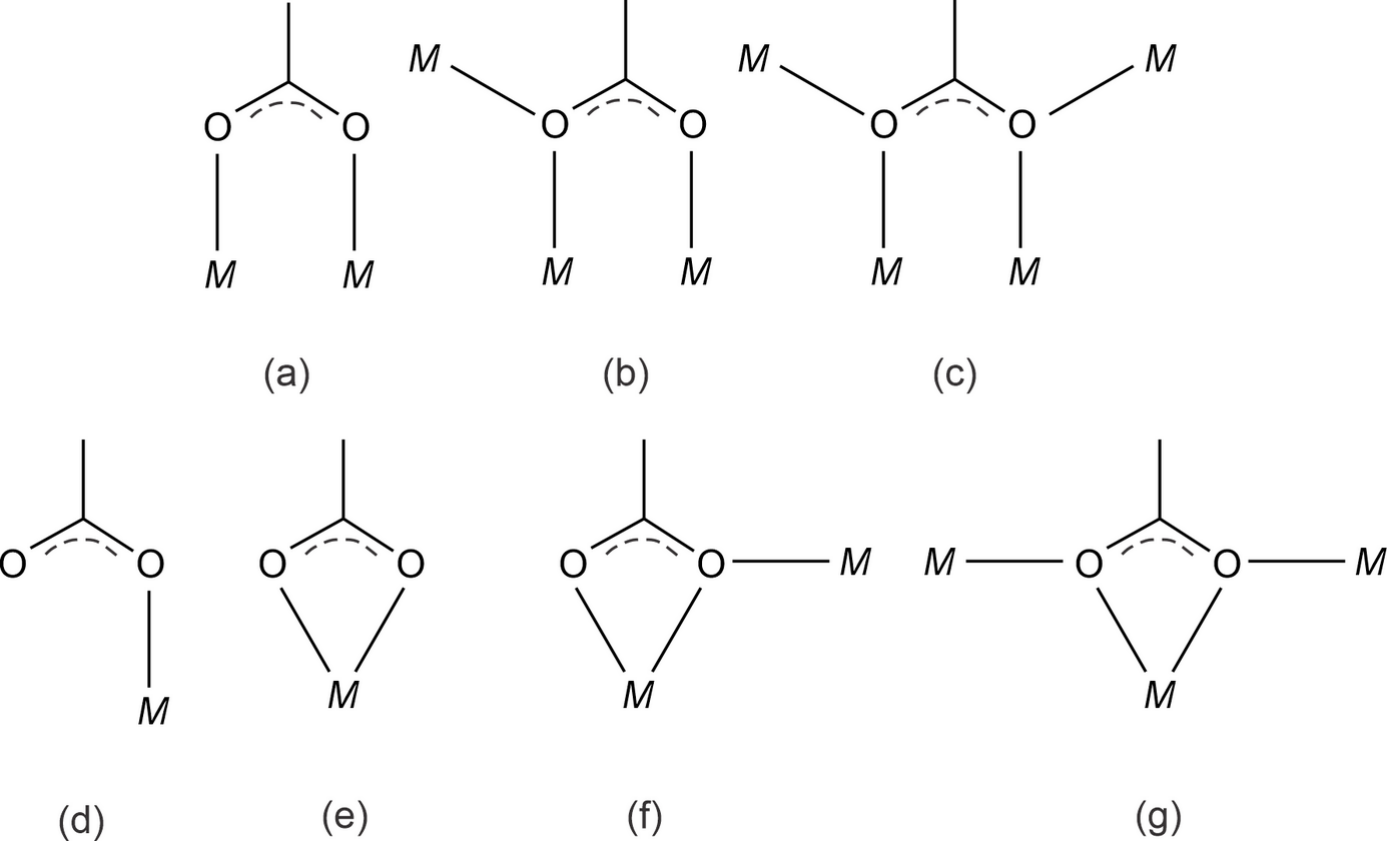
Possible coordination modes of the acetato ligand to metal cations *M*.

From the seven different possible coordination modes of acetato ligands to central *M*^II^ cations shown in Fig. 2 ▸, the acetate groups in the structure of the title compound feature only three. Coordination mode (*a*) is bridging two Ni^II^ cations in a bis-monodentate manner, μ_2_-(-κ^1^*O*,κ^1^*O*′), and realized for carboxyl­ate group C2(O1)O2; mode (*b*) is bridging three Ni^II^ cations in a monodentate-bis-monodentate manner, μ_3_-(-κ^1^*O*κ^2^*O*′), and realized for carboxyl­ate groups C4(O3)O4 and C7(O8)O7; mode (*c*) is bridging four Ni^II^ cations in a bis­(bis-monodentate) manner, μ_4_-(-κ^2^*O*κ^2^*O*′, and realized for carboxyl­ate group C5(O5)O6. Monodentate coordination mode (*d*), or any of the chelating coordination modes (*e*–*g*) detailed in Fig. 2 ▸ are not realized, but are known for other divalent first-row transition metals *M*, *e.g.* for anhydrous iron(II) acetate (Weber *et al.*, 2011 ▸). The oxygen atom of the hy­droxy group, O9H9, bridges three Ni^II^ cations (Ni2, Ni3, Ni3′). Together with the attached H9 atom, the environment of O9 is distorted tetra­hedral, with Ni—O—Ni angles ranging from 97.13 (8) to 121.18 (9)°.

**Figure 3:**
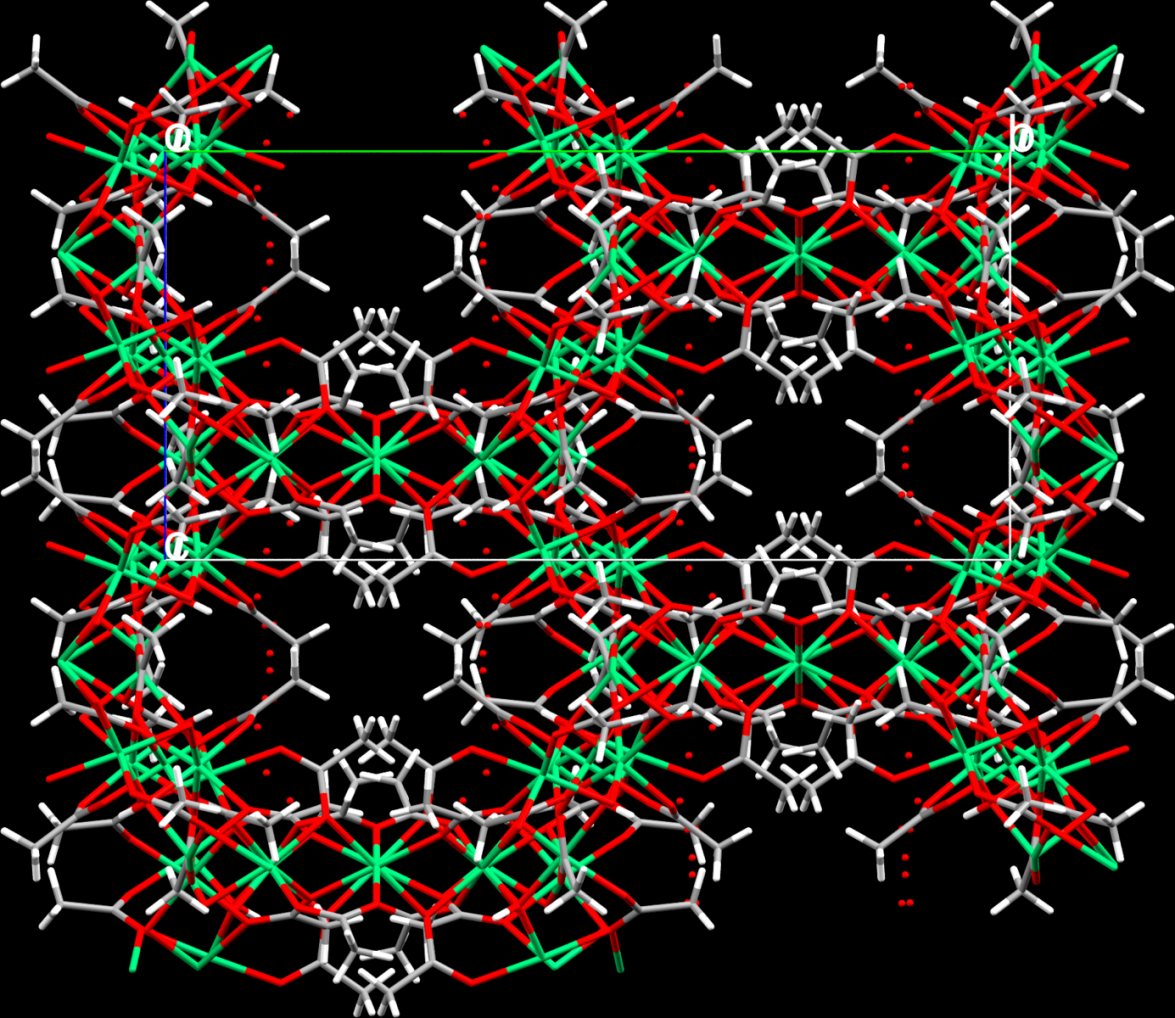
Packing plot of [Ni_5_(OAc)_8_(OH)_2_]·2.60H_2_O along [100].

**Figure 4:**
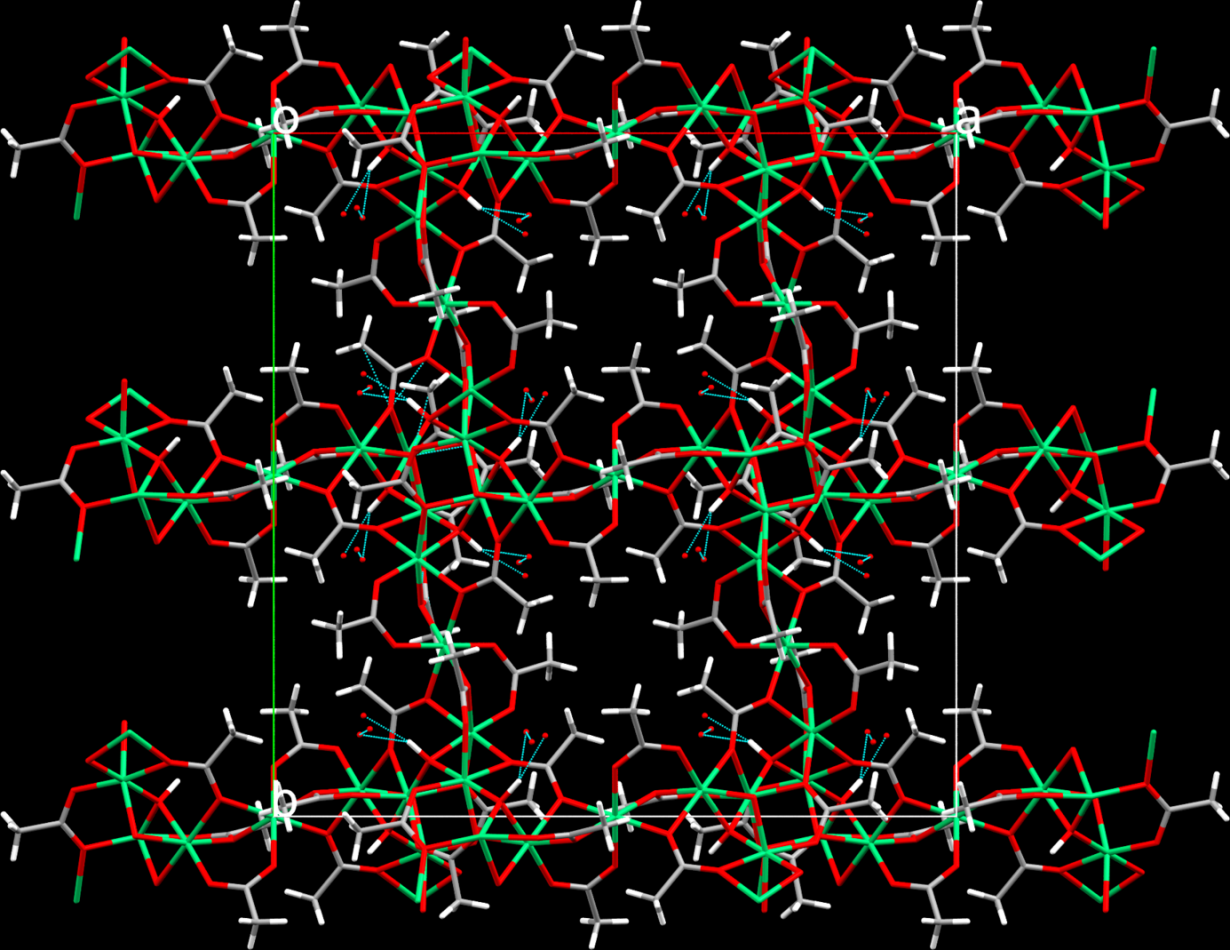
Packing plot of [Ni_5_(OAc)_8_(OH)_2_]·2.60H_2_O along [001] with hydrogen-bonding inter­actions between the hy­droxy group and the O atoms of disordered water mol­ecules (shown as blue dotted lines).

The μ_2_- μ_3_-, μ_4_- and μ_3_-bridging modes of the acetato ligands and the μ_3_-mode of the OH group, respectively, lead to the formation of a framework structure, whereby the arrangement of the acetato ligands with the methyl groups pointing away from the Ni^II^ cations creates hydro­phobic channels extending parallel to the main crystallographic axes (Figs. 3 ▸, 4 ▸). The disordered water mol­ecules of crystallization are situated in pockets near to the hy­droxy group to which they are hydrogen-bonded (Table 2 ▸, Fig. 4 ▸). In addition, typical donor⋯acceptor distances suitable for hydrogen bonds of moderate strength are present between O7⋯O*W*2 and O2*W*⋯O3*W* (Table 2 ▸). These inter­actions might further consolidate the crystal structure.

## Synthesis and crystallization

Single crystals of [Ni_5_(OAc)_8_(OH)_2_]·2.60H_2_O were inadvertently obtained by reacting Ni(OAc)_2_·4H_2_O, KOH (>85%_wt_) and ∼80%_wt_ H_3_AsO_4_ under hydro­thermal conditions in an approximate 3:2:3 molar ratio. The reactants were introduced in a Teflon lined steal autoclave and heated at 493 K for 3 d. After cooling to room temperature, large faint greenish plates of [Ni_5_(OAc)_8_(OH)_2_]·2.60H_2_O were directly isolated from the mother liquor under a polarizing microscope.

## Refinement

Crystal data, data collection and structure refinement details are summarized in Table 3 ▸. The H atom of the hydroxide group was located in a difference-Fourier map and was refined as riding on the parent O atom with *U*_iso_(H) = 1.5*U*_eq_(O). Free refinement of the occupation factors of the three crystal water O-atom positions indicated underoccupation for all of them. For the final structure model, the two least occupied positions (O*W*2, O*W*3) were paired and coupled with the occupation factor of the most occupied site (O*W*1) so that the sum of site occupation factors equals 1. The water H atoms could not be located and were excluded from the structural model, but are included for calculation of crystal data.

## Supplementary Material

Crystal structure: contains datablock(s) I. DOI: 10.1107/S2414314625000823/bt4163sup1.cif

Structure factors: contains datablock(s) I. DOI: 10.1107/S2414314625000823/bt4163Isup2.hkl

CCDC reference: 2420345

Additional supporting information:  crystallographic information; 3D view; checkCIF report

## Figures and Tables

**Table 1:** Selected bond lengths (Å)

Ni1—O2	1.993 (2)	Ni2—O6^ii^	2.1140 (18)
Ni1—O3	2.1037 (18)	Ni3—O9	1.9747 (18)
Ni1—O5	2.1257 (19)	Ni3—O9^i^	1.9902 (17)
Ni2—O1	1.993 (2)	Ni3—O7	2.0474 (19)
Ni2—O9	1.9962 (18)	Ni3—O4^i^	2.0750 (19)
Ni2—O3	2.0698 (18)	Ni3—O8^i^	2.1023 (19)
Ni2—O5	2.0937 (19)	Ni3—O6	2.1259 (18)
Ni2—O8^i^	2.1016 (18)		

Symmetry codes: (i) 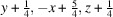; (ii) 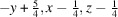.

**Table 2:** Hydrogen-bond geometry (Å, °)

*D*—H⋯*A*	*D*—H	H⋯*A*	*D*⋯*A*	*D*—H⋯*A*
O9—H9⋯O*W*1	1.00	2.06	2.957 (4)	147
O9—H9⋯O*W*3	1.00	1.85	2.827 (8)	166
O7⋯O*W*2^ii^			2.87	
O*W*2⋯O*W*3			2.85	

Symmetry code: (ii) 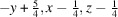.

**Table 3:** Experimental details

Crystal data	
Chemical formula	[Ni_5_(C_2_H_3_O_2_)_8_(OH)_2_]·2.60H2O
*M*_r_	846.80
Crystal system, space group	Tetragonal, *I*4_1_/*a*
Temperature (K)	100
*a*, *c* (Å)	23.3025 (11), 11.2648 (5)
*V* (Å^3^)	6116.9 (6)
*Z*	8
Radiation type	Mo *K*α
μ (mm^−1^)	3.10
Crystal size (mm)	0.20 × 0.12 × 0.05 × 0.04 (radius)

Data collection	
Diffractometer	Stoe Stadivari
Absorption correction	Multi-scan (*LANA*; Koziskova *et al.*, 2016 ▸)
*T*_min_, *T*_max_	0.581, 0.710
No. of measured, independent and observed [*I* > 2σ(*I*)] reflections	20999, 5145, 2744
*R*_int_	0.069
(sin θ/λ)_max_ (Å^−1^)	0.756

Refinement	
*R*[*F*^2^ > 2σ(*F*^2^)], *wR*(*F*^2^), *S*	0.038, 0.069, 0.83
No. of reflections	5145
No. of parameters	210
H-atom treatment	H-atom parameters constrained
Δρ_max_, Δρ_min_ (e Å^−3^)	0.53, −0.91

Computer programs: *X-AREA* (Stoe & Cie, 2024 ▸), *SHELXT* (Sheldrick, 2015*a* ▸), *SHELXL* (Sheldrick, 2015*b* ▸), *Mercury* (Macrae *et al.*, 2020 ▸) and *publCIF* (Westrip, 2010 ▸).

## full crystallographic data

### Poly[[bis(µ4-acetato-κ4O:O:O':O')tetrakis(µ3-acetato-κ3O:O:O')bis(µ2-acetato-κ2O:O')bis(µ3-hydroxido)pentanickel(II)] 2.60-hydrate] Crystal data

**Table:**

[Ni_5_(C_2_H_3_O_2_)_8_(OH)_2_]·2.60H2O	*D*_x_ = 1.839 Mg m^−^^3^
*M**_r_* = 846.80	Mo *K*α radiation, λ = 0.71073 Å
Tetragonal, *I*4_1_/*a*	Cell parameters from 9821 reflections
*a* = 23.3025 (11) Å	θ = 1.8–30.9°
*c* = 11.2648 (5) Å	µ = 3.10 mm^−^^1^
*V* = 6116.9 (6) Å^3^	*T* = 100 K
*Z* = 8	Plate, light green
*F*(000) = 3456	0.20 × 0.12 × 0.05 × 0.04 (radius) mm

### Poly[[bis(µ4-acetato-κ4O:O:O':O')tetrakis(µ3-acetato-κ3O:O:O')bis(µ2-acetato-κ2O:O')bis(µ3-hydroxido)pentanickel(II)] 2.60-hydrate] Data collection

**Table:**

Stoe Stadivari diffractometer	5145 independent reflections
Radiation source: Axo_Mo	2744 reflections with *I* > 2σ(*I*)
Graded multilayer mirror monochromator	*R*_int_ = 0.069
Detector resolution: 13.33 pixels mm^-1^	θ_max_ = 32.5°, θ_min_ = 2.0°
rotation method, ω scans	*h* = −31→35
Absorption correction: multi-scan (*LANA*; Koziskova *et al*., 2016)	*k* = −34→33
*T*_min_ = 0.581, *T*_max_ = 0.710	*l* = −16→7
20999 measured reflections	

### Poly[[bis(µ4-acetato-κ4O:O:O':O')tetrakis(µ3-acetato-κ3O:O:O')bis(µ2-acetato-κ2O:O')bis(µ3-hydroxido)pentanickel(II)] 2.60-hydrate] Refinement

**Table:**

Refinement on *F*^2^	0 restraints
Least-squares matrix: full	Hydrogen site location: inferred from neighbouring sites
*R*[*F*^2^ > 2σ(*F*^2^)] = 0.038	H-atom parameters constrained
*wR*(*F*^2^) = 0.069	*w* = 1/[σ^2^(*F*_o_^2^) + (0.026*P*)^2^] where *P* = (*F*_o_^2^ + 2*F*_c_^2^)/3
*S* = 0.83	(Δ/σ)_max_ = 0.002
5145 reflections	Δρ_max_ = 0.53 e Å^−^^3^
210 parameters	Δρ_min_ = −0.91 e Å^−^^3^

### Poly[[bis(µ4-acetato-κ4O:O:O':O')tetrakis(µ3-acetato-κ3O:O:O')bis(µ2-acetato-κ2O:O')bis(µ3-hydroxido)pentanickel(II)] 2.60-hydrate] Special details

**Table:**

Geometry. All esds (except the esd in the dihedral angle between two l.s. planes) are estimated using the full covariance matrix. The cell esds are taken into account individually in the estimation of esds in distances, angles and torsion angles; correlations between esds in cell parameters are only used when they are defined by crystal symmetry. An approximate (isotropic) treatment of cell esds is used for estimating esds involving l.s. planes.

### Poly[[bis(µ4-acetato-κ4O:O:O':O')tetrakis(µ3-acetato-κ3O:O:O')bis(µ2-acetato-κ2O:O')bis(µ3-hydroxido)pentanickel(II)] 2.60-hydrate] Fractional atomic coordinates and isotropic or equivalent isotropic displacement parameters (Å^2^)

**Table:**

	*x*	*y*	*z*	*U*_iso_*/*U*_eq_	Occ. (<1)
Ni1	1.000000	0.500000	0.500000	0.01941 (13)	
Ni2	0.87305 (2)	0.53890 (2)	0.49712 (3)	0.01354 (8)	
Ni3	0.77923 (2)	0.44682 (2)	0.52555 (3)	0.01217 (8)	
O1	0.90748 (9)	0.59881 (9)	0.60134 (16)	0.0211 (5)	
O2	1.00011 (9)	0.57249 (10)	0.59387 (17)	0.0284 (5)	
O3	0.94065 (8)	0.53418 (9)	0.37833 (14)	0.0183 (5)	
O4	0.88695 (8)	0.53130 (9)	0.21325 (15)	0.0214 (5)	
O5	0.92122 (8)	0.47408 (9)	0.57859 (15)	0.0191 (5)	
O6	0.84795 (8)	0.42100 (8)	0.63759 (14)	0.0134 (4)	
O7	0.70649 (8)	0.46180 (8)	0.42844 (15)	0.0178 (4)	
O8	0.72147 (8)	0.54712 (8)	0.34548 (14)	0.0136 (4)	
O9	0.83183 (8)	0.47852 (8)	0.40476 (14)	0.0122 (4)	
H9	0.858792	0.448659	0.373873	0.018*	
OW1	0.8698 (2)	0.37614 (17)	0.2711 (4)	0.0573 (14)	0.699 (5)
OW2	0.8781 (4)	0.3908 (4)	0.0907 (8)	0.070 (4)	0.301 (5)
OW3	0.8977 (4)	0.3816 (4)	0.3396 (8)	0.041 (3)	0.301 (5)
C1	0.97488 (17)	0.65457 (18)	0.7052 (4)	0.0710 (15)	
H1A	0.965806	0.690456	0.663914	0.106*	
H1B	1.015970	0.653458	0.723552	0.106*	
H1C	0.952762	0.652406	0.779061	0.106*	
C2	0.95967 (16)	0.60452 (16)	0.6271 (3)	0.0316 (8)	
C3	0.98514 (14)	0.51384 (18)	0.1903 (3)	0.0435 (11)	
H3A	0.994597	0.547377	0.141613	0.065*	
H3B	0.976865	0.481045	0.138644	0.065*	
H3C	1.017715	0.504596	0.241930	0.065*	
C4	0.93369 (12)	0.52682 (13)	0.2648 (2)	0.0205 (6)	
C5	0.94067 (13)	0.37866 (14)	0.6475 (3)	0.0338 (9)	
H5A	0.938084	0.369374	0.732144	0.051*	
H5B	0.980102	0.389891	0.628096	0.051*	
H5C	0.929924	0.344947	0.600447	0.051*	
C6	0.90074 (13)	0.42727 (13)	0.6195 (2)	0.0183 (7)	
C7	0.62615 (14)	0.52147 (16)	0.4064 (3)	0.0487 (11)	
H7A	0.618561	0.561801	0.387364	0.073*	
H7B	0.614544	0.513707	0.488435	0.073*	
H7C	0.604258	0.496792	0.352400	0.073*	
C8	0.68878 (12)	0.50943 (13)	0.3925 (2)	0.0167 (6)	

### Poly[[bis(µ4-acetato-κ4O:O:O':O')tetrakis(µ3-acetato-κ3O:O:O')bis(µ2-acetato-κ2O:O')bis(µ3-hydroxido)pentanickel(II)] 2.60-hydrate] Atomic displacement parameters (Å^2^)

**Table:**

	*U*^11^	*U*^22^	*U*^33^	*U*^12^	*U*^13^	*U*^23^
Ni1	0.0095 (3)	0.0283 (3)	0.0204 (3)	−0.0013 (2)	−0.0010 (2)	0.0099 (2)
Ni2	0.01025 (19)	0.0152 (2)	0.01523 (16)	−0.00078 (14)	−0.00080 (15)	0.00298 (15)
Ni3	0.0127 (2)	0.01059 (19)	0.01326 (15)	−0.00031 (14)	0.00098 (14)	0.00057 (14)
O1	0.0166 (12)	0.0224 (13)	0.0244 (10)	−0.0019 (9)	−0.0067 (9)	−0.0002 (9)
O2	0.0157 (13)	0.0348 (15)	0.0348 (12)	−0.0056 (10)	−0.0080 (10)	0.0042 (11)
O3	0.0107 (11)	0.0305 (13)	0.0138 (9)	−0.0011 (9)	−0.0005 (8)	0.0093 (9)
O4	0.0134 (11)	0.0343 (14)	0.0165 (9)	0.0008 (9)	−0.0017 (8)	0.0020 (9)
O5	0.0110 (11)	0.0244 (13)	0.0219 (10)	−0.0022 (9)	−0.0023 (8)	0.0117 (9)
O6	0.0107 (11)	0.0138 (11)	0.0157 (9)	−0.0002 (8)	0.0016 (8)	0.0037 (8)
O7	0.0201 (12)	0.0142 (11)	0.0192 (9)	−0.0033 (9)	−0.0056 (9)	0.0021 (9)
O8	0.0114 (11)	0.0141 (11)	0.0154 (8)	−0.0020 (8)	0.0005 (8)	0.0001 (8)
O9	0.0125 (11)	0.0103 (10)	0.0138 (8)	0.0021 (8)	0.0014 (8)	0.0005 (8)
OW1	0.062 (3)	0.050 (3)	0.060 (3)	0.013 (2)	0.009 (3)	0.001 (2)
OW2	0.057 (8)	0.087 (9)	0.065 (6)	0.037 (6)	−0.022 (5)	−0.016 (6)
OW3	0.044 (6)	0.033 (5)	0.047 (5)	0.016 (4)	−0.029 (4)	−0.020 (4)
C1	0.049 (3)	0.060 (3)	0.104 (3)	0.001 (2)	−0.044 (3)	−0.038 (3)
C2	0.034 (2)	0.031 (2)	0.0298 (18)	−0.0104 (17)	−0.0141 (16)	0.0046 (15)
C3	0.019 (2)	0.087 (3)	0.0240 (16)	0.0062 (19)	0.0037 (15)	0.0094 (19)
C4	0.0133 (15)	0.0274 (18)	0.0208 (14)	0.0023 (12)	0.0019 (13)	0.0057 (13)
C5	0.0184 (19)	0.036 (2)	0.0472 (19)	0.0124 (15)	0.0106 (16)	0.0220 (17)
C6	0.0160 (17)	0.0234 (18)	0.0154 (13)	0.0011 (13)	0.0001 (12)	0.0036 (12)
C7	0.015 (2)	0.056 (3)	0.075 (3)	0.0044 (18)	0.0072 (19)	0.044 (2)
C8	0.0114 (15)	0.0257 (18)	0.0129 (12)	0.0006 (13)	−0.0030 (11)	0.0013 (12)

### Poly[[bis(µ4-acetato-κ4O:O:O':O')tetrakis(µ3-acetato-κ3O:O:O')bis(µ2-acetato-κ2O:O')bis(µ3-hydroxido)pentanickel(II)] 2.60-hydrate] Geometric parameters (Å, º)

**Table:**

Ni1—O2^i^	1.993 (2)	O5—C6	1.277 (3)
Ni1—O2	1.993 (2)	O6—C6	1.255 (3)
Ni1—O3^i^	2.1037 (18)	O7—C8	1.251 (3)
Ni1—O3	2.1037 (18)	O8—C8	1.278 (3)
Ni1—O5	2.1257 (19)	O9—H9	1.0000
Ni1—O5^i^	2.1257 (19)	C1—H1A	0.9800
Ni2—O1	1.993 (2)	C1—H1B	0.9800
Ni2—O9	1.9962 (18)	C1—H1C	0.9800
Ni2—O3	2.0698 (18)	C1—C2	1.503 (5)
Ni2—O5	2.0937 (19)	C3—H3A	0.9800
Ni2—O8^ii^	2.1016 (18)	C3—H3B	0.9800
Ni2—O6^iii^	2.1140 (18)	C3—H3C	0.9800
Ni3—O9	1.9747 (18)	C3—C4	1.494 (4)
Ni3—O9^ii^	1.9902 (17)	C5—H5A	0.9800
Ni3—O7	2.0474 (19)	C5—H5B	0.9800
Ni3—O4^ii^	2.0750 (19)	C5—H5C	0.9800
Ni3—O8^ii^	2.1023 (19)	C5—C6	1.499 (4)
Ni3—O6	2.1259 (18)	C7—H7A	0.9800
O1—C2	1.257 (4)	C7—H7B	0.9800
O2—C2	1.259 (4)	C7—H7C	0.9800
O3—C4	1.301 (3)	C7—C8	1.495 (4)
O4—C4	1.238 (3)		
			
O2^i^—Ni1—O2	180.0	C6—O5—Ni1	135.72 (19)
O2—Ni1—O3	91.47 (8)	C6—O5—Ni2	125.03 (18)
O2—Ni1—O3^i^	88.53 (8)	Ni2^ii^—O6—Ni3	89.64 (7)
O2^i^—Ni1—O3	88.53 (8)	C6—O6—Ni2^ii^	142.11 (18)
O2^i^—Ni1—O3^i^	91.47 (8)	C6—O6—Ni3	127.48 (17)
O2—Ni1—O5	91.20 (8)	C8—O7—Ni3	126.65 (19)
O2—Ni1—O5^i^	88.80 (8)	Ni2^iii^—O8—Ni3^iii^	94.22 (7)
O2^i^—Ni1—O5^i^	91.20 (8)	C8—O8—Ni2^iii^	136.79 (19)
O2^i^—Ni1—O5	88.80 (8)	C8—O8—Ni3^iii^	123.99 (18)
O3—Ni1—O3^i^	180.0	Ni2—O9—H9	111.7
O3^i^—Ni1—O5	100.88 (7)	Ni3—O9—Ni2	101.72 (7)
O3—Ni1—O5	79.12 (7)	Ni3^iii^—O9—Ni2	97.13 (8)
O3^i^—Ni1—O5^i^	79.12 (7)	Ni3—O9—Ni3^iii^	121.18 (9)
O3—Ni1—O5^i^	100.89 (7)	Ni3—O9—H9	111.7
O5—Ni1—O5^i^	180.0	Ni3^iii^—O9—H9	111.7
O1—Ni2—O3	96.42 (8)	H1A—C1—H1B	109.5
O1—Ni2—O5	91.80 (8)	H1A—C1—H1C	109.5
O1—Ni2—O6^iii^	94.78 (7)	H1B—C1—H1C	109.5
O1—Ni2—O8^ii^	96.25 (8)	C2—C1—H1A	109.5
O1—Ni2—O9	174.04 (8)	C2—C1—H1B	109.5
O3—Ni2—O5	80.62 (7)	C2—C1—H1C	109.5
O3—Ni2—O6^iii^	91.54 (7)	O1—C2—O2	126.4 (3)
O3—Ni2—O8^ii^	167.29 (7)	O1—C2—C1	116.4 (3)
O5—Ni2—O6^iii^	170.29 (7)	O2—C2—C1	117.2 (3)
O5—Ni2—O8^ii^	97.89 (7)	H3A—C3—H3B	109.5
O8^ii^—Ni2—O6^iii^	88.49 (7)	H3A—C3—H3C	109.5
O9—Ni2—O3	89.53 (7)	H3B—C3—H3C	109.5
O9—Ni2—O5	88.74 (7)	C4—C3—H3A	109.5
O9—Ni2—O6^iii^	85.46 (7)	C4—C3—H3B	109.5
O9—Ni2—O8^ii^	77.80 (7)	C4—C3—H3C	109.5
O4^ii^—Ni3—O6	85.24 (7)	O3—C4—C3	118.6 (3)
O4^ii^—Ni3—O8^ii^	166.95 (8)	O4—C4—O3	124.0 (3)
O7—Ni3—O4^ii^	89.94 (8)	O4—C4—C3	117.4 (2)
O7—Ni3—O6	171.54 (7)	H5A—C5—H5B	109.5
O7—Ni3—O8^ii^	102.12 (7)	H5A—C5—H5C	109.5
O8^ii^—Ni3—O6	83.37 (7)	H5B—C5—H5C	109.5
O9^ii^—Ni3—O4^ii^	86.88 (7)	C6—C5—H5A	109.5
O9—Ni3—O4^ii^	95.99 (7)	C6—C5—H5B	109.5
O9—Ni3—O6	92.72 (7)	C6—C5—H5C	109.5
O9^ii^—Ni3—O6	85.30 (7)	O5—C6—C5	119.3 (3)
O9—Ni3—O7	94.70 (7)	O6—C6—O5	121.6 (3)
O9^ii^—Ni3—O7	87.50 (7)	O6—C6—C5	119.1 (3)
O9—Ni3—O8^ii^	78.25 (7)	H7A—C7—H7B	109.5
O9^ii^—Ni3—O8^ii^	98.49 (7)	H7A—C7—H7C	109.5
O9—Ni3—O9^ii^	176.38 (9)	H7B—C7—H7C	109.5
C2—O1—Ni2	126.8 (2)	C8—C7—H7A	109.5
C2—O2—Ni1	131.2 (2)	C8—C7—H7B	109.5
Ni2—O3—Ni1	95.69 (7)	C8—C7—H7C	109.5
C4—O3—Ni1	132.29 (19)	O7—C8—O8	123.2 (3)
C4—O3—Ni2	123.22 (18)	O7—C8—C7	117.0 (3)
C4—O4—Ni3^iii^	131.83 (17)	O8—C8—C7	119.8 (3)
Ni2—O5—Ni1	94.33 (7)		
			
Ni1—O2—C2—O1	−4.1 (5)	Ni2^ii^—O6—C6—O5	−150.4 (2)
Ni1—O2—C2—C1	175.8 (2)	Ni2^ii^—O6—C6—C5	29.1 (5)
Ni1—O3—C4—O4	151.5 (2)	Ni2^iii^—O8—C8—O7	166.11 (17)
Ni1—O3—C4—C3	−30.4 (4)	Ni2^iii^—O8—C8—C7	−13.8 (4)
Ni1—O5—C6—O6	−164.02 (18)	Ni3^iii^—O4—C4—O3	−18.6 (5)
Ni1—O5—C6—C5	16.4 (4)	Ni3^iii^—O4—C4—C3	163.2 (2)
Ni2—O1—C2—O2	−1.8 (5)	Ni3—O6—C6—O5	43.1 (4)
Ni2—O1—C2—C1	178.3 (2)	Ni3—O6—C6—C5	−137.4 (2)
Ni2—O3—C4—O4	12.4 (4)	Ni3—O7—C8—O8	44.2 (3)
Ni2—O3—C4—C3	−169.5 (2)	Ni3—O7—C8—C7	−135.9 (2)
Ni2—O5—C6—O6	−15.7 (4)	Ni3^iii^—O8—C8—O7	18.2 (4)
Ni2—O5—C6—C5	164.8 (2)	Ni3^iii^—O8—C8—C7	−161.7 (2)

Symmetry codes: (i) −*x*+2, −*y*+1, −*z*+1; (ii) *y*+1/4, −*x*+5/4, *z*+1/4; (iii) −*y*+5/4, *x*−1/4, *z*−1/4.

### Poly[[bis(µ4-acetato-κ4O:O:O':O')tetrakis(µ3-acetato-κ3O:O:O')bis(µ2-acetato-κ2O:O')bis(µ3-hydroxido)pentanickel(II)] 2.60-hydrate] Hydrogen-bond geometry (Å, º)

**Table:**

*D*—H···*A*	*D*—H	H···*A*	*D*···*A*	*D*—H···*A*
O9—H9···O*W*1	1.00	2.06	2.957 (4)	147
O9—H9···O*W*3	1.00	1.85	2.827 (8)	166
O7···O*W*2^iii^			2.87	
O*W*2···O*W*3			2.85	

Symmetry code: (iii) −*y*+5/4, *x*−1/4, *z*−1/4.
